# Quantitative Analysis of Dynamic Subacromial Ultrasonography: Reliability and Influencing Factors

**DOI:** 10.3389/fbioe.2022.830508

**Published:** 2022-02-15

**Authors:** Che-Yu Lin, Chia-Ching Chou, Lan-Rong Chen, Wei-Ting Wu, Po-Cheng Hsu, Tung-Han Yang, Ke-Vin Chang

**Affiliations:** ^1^ Institute of Applied Mechanics, College of Engineering, National Taiwan University, Taipei, Taiwan; ^2^ Department of Physical Medicine and Rehabilitation and Community and Geriatric Research Center, National Taiwan University Hospital, Bei-Hu Branch, Taipei, Taiwan; ^3^ Department of Physical Medicine and Rehabilitation, National Taiwan University College of Medicine, Taipei, Taiwan; ^4^ Department of Physical Medicine and Rehabilitation, Taipei Veterans General Hospital, Taipei, Taiwan; ^5^ Center for Regional Anesthesia and Pain Medicine, Wang-Fang Hospital, Taipei Medical University, Taipei, Taiwan

**Keywords:** ultrasound, subacrommial impingement, sport, rehabilitation, pain

## Abstract

**Objective:** Current imaging methods used to examine patients with subacromial impingement syndrome (SIS) are limited by their semi-quantitative nature and their capability of capturing dynamic movements. This study aimed to develop a quantitative analytic model to assess subacromial motions using dynamic ultrasound and to examine their reliability and potential influencing factors.

**Method:** We included 48 healthy volunteers and examined their subacromial motions with dynamic ultrasound imaging. The parameters were the minimal vertical acromiohumeral distance, rotation radius, and degrees of the humeral head. The generalized estimating equation (GEE) was used to investigate the impact of different shoulder laterality, postures, and motion phases on the outcome.

**Result:** Using the data of the minimal vertical acromiohumeral distance, the intra-rater and inter-rater reliabilities (intra-class correlation coefficient) were determined as 0.94 and 0.88, respectively. In the GEE analysis, a decrease in the minimal vertical acromiohumeral distance was associated with the abduction phase and full-can posture, with a beta coefficient of −0.02 cm [95% confidence interval (CI), −0.03 to −0.01] and −0.07 cm (95% CI, −0.11 to −0.02), respectively. The abduction phase led to a decrease in the radius of humeral rotation and an increase in the angle of humeral rotation, with a beta coefficient of −1.28 cm (95% CI, −2.16 to −0.40) and 6.60° (95% CI, 3.54–9.67), respectively. A significant negative correlation was observed between the rotation angle and radius of the humeral head and between the rotation angle and the minimal vertical acromiohumeral distance.

**Conclusion:** Quantitative analysis of dynamic ultrasound imaging enables the delineation of subacromial motion with good reliability. The vertical acromiohumeral distance is the lowest in the abduction phase and full-can posture, and the rotation angle of the humeral head has the potential to serve as a new parameter for the evaluation of SIS.

## Introduction

Subaromial impingement syndrome (SIS) is the most common cause of shoulder pain, with a reported prevalence of 48% in a survey of 35,150 patients with shoulder complaints (van der Windt et al., 1995). The incidence of SIS is high among athletes with repetitive overhead activities of the arm, such as volleyball players and swimmers (Lo et al., 1990). Various physical tests have been applied for the diagnosis of SIS, such as the painful arc, Neer’s impingement, and Hawkins-Kennedy tests, but their sensitivity and specificity are mostly unsatisfactory (Chang K.-V. et al., 2020). Owing to the limitations of physical examinations, several imaging methods have been developed to better assess SIS. In 2018, Cunningham et al. measured the angle of the greater tuberosity in relation to the center of rotation of the humeral head on radiographs and found that an increase in the aforementioned angle was associated with rotator cuff tendon tears (Cunningham et al., 2018). In 2019, Kenmoku et al. used magnetic resonance imaging (MRI) to evaluate 73 asymptomatic shoulders and 110 shoulders with SIS, revealing that shoulders with SIS had significant restriction of glenohumeral rotation (Kenmoku et al., 2019). However, the two imaging tools mentioned above are not widely used for the evaluation of SIS, considering their accessibility, cost-effectiveness, portability, and capability of dynamic assessment.

In recent years, ultrasound has emerged as the first choice for analyzing musculoskeletal disorders (Chang P.-H. et al., 2020; Wu et al., 2021), including SIS, based on its high resolution on superficial soft tissues and the allowance of real-time imaging. Its capability in delineating rotator cuff disorders has been demonstrated to be comparable to that of MRI (Roy et al., 2015). Until now, there have been two major types of dynamic ultrasound methods for SIS assessment. The first is used to measure the acromiohumeral distance at fixed angles of arm abduction (de Oliveira et al., 2020). The second is used to observe the deformation of the subacromial soft tissue or reciprocal position of the humeral head in relation to the acromion during shoulder elevation (Bureau et al., 2006). The main disadvantage of the first approach is that the acromiohumeral distance determined under static conditions (such as 0° and 60° of shoulder abduction) is incapable of reproducing abnormalities seen during shoulder movements (de Oliveira et al., 2020). However, although the second approach allows the detection of uncoordinated subacromial motion (Bureau et al., 2006), its application in clinical assessment and follow-up is limited by the semi-quantitative nature of the grading scenario (Chang et al., 2016; Chang et al., 2017). In this regard, this study aimed to develop quantitative indicators of subacromial motions using dynamic ultrasound imaging and to examine their reliability and potential influencing factors.

## Materials and Methods

### Participants

As the present study was the first attempt to validate the methodology of subacromial reciprocal movement quantification, we included only participants without any shoulder symptoms who visited the department of physical medicine and rehabilitation. The inclusion criteria were as follows: 1) > 20 years of age, 2) capable of completing a questionnaire, 3) denying any shoulder discomfort, 4) with no limitation of range of shoulder motion, and 5) without antecedent surgeries and interventions on the shoulder regions. The exclusion criteria were as follows: 1) cognitive impairment, 2) active medical condition (e.g., unstable angina), 3) known neuromuscular disorders that were likely to affect muscle strength (e.g., stroke and myasthenia gravis), and 4) rotator cuff tendon tears identified on ultrasound imaging. A total of 48 people were recruited and divided into six subgroups based on the stratification of the differences in sex and age. The institutional review board of the hospital approved the research proposal (IRB No. 201910036RINC), and written informed consent was obtained from all participants prior to formal enrollment.

### Evaluation of Shoulder Symptoms and Function

The Shoulder Pain and Disability Index (SPADI) was used to evaluate shoulder symptoms and function, comprising two subscales with 13 items: pain (five items) and disability (eight items) (Yao et al., 2017). Each item is scored from 0 (no pain/disability) to 10 (maximal pain/disability), and their summation is then transformed to a 100-point scale. In the present study, the participants were required to report a score of 0 for their bilateral shoulders on the SPADI score.

### Shoulder Ultrasound Examination

Scout scanning was performed for the long head of the biceps tendon, subscapularis tendon, supraspinatus tendon, and infraspinatus tendon. The subacromial-subdeltoid bursa was examined though the short-axis view for the supraspinatus tendon with the shoulder in internal rotation. It was shown as a hypoechoic stripe interposed between the superficial and deep hyperechoic peribursal fat. The thickness of the subacromial-subdeltoid bursa was defined as the depth of the aforementioned hypoechoic stripe in addition to the superficial peribursal fat and a value of >2 mm was considered abnormal (Chang et al., 2017). The diagnostic criteria of pathology were based on previous literature (Han et al., 2021; Wu et al., 2021) and patients with tears of any of the aforementioned tendons were excluded from this study.

**FIGURE 1:**
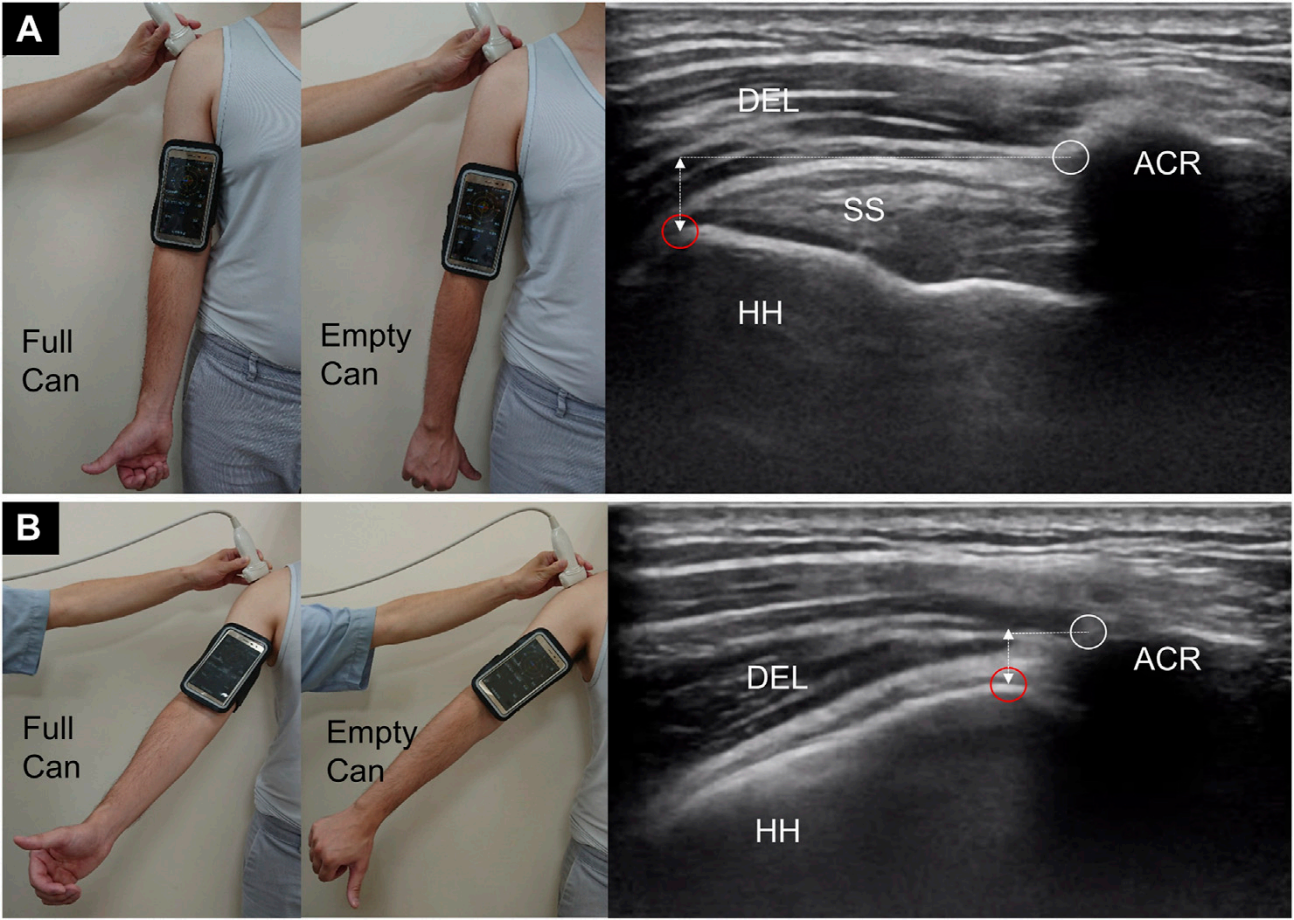
The posture of the upper extremity and ultrasound imaging of the subacromial region at the starting **(A)** and abducted **(B)** position. In **(B)**, the greater tuberosity (red circles) is about to pass the lateral acromial edge (white circles). DEL, deltoid muscle; HH, humeral head; SS, supraspinatus tendon; ACR, acromion; double-head dashed line, vertical acromiohumeral distance.

During the dynamic examination, the participants were seated with both arms naturally positioned beside the trunk (Figure 1A). A smartphone was secured on the arm being examined distal to the insertion of the middle deltoid muscle. The application software, GPS Status and toolbox version 8.4.177 (Hungary, 1,033 Budapest, Hévízi u. 5.) (Chang et al., 2019), was installed on the smartphone to measure the angle of abduction during arm elevation. A linear ultrasound transducer (5–18 MHz; HI VISION Ascendus, Hitachi) was placed along the scapular plane with its mid-point on the lateral edge of the acromion, where the humeral head, supraspinatus tendon, and acromion could be clearly visualized. The participants were invited to actively abduct the arm to the level where the greater tuberosity had rotated to a position underneath the acromion (Figure 1B) followed by a natural return to the initial position. They were suggested to raise and drop their arms at a speed while attempting to reach an overhead object.

During the dynamic test, there were two postures: one with the thumb pointing upward (full-can posture) and the other with the thumb pointing downward with the arm internally rotated (empty-can posture). The participants were asked to abduct and adduct the arm for five repetitions in each posture. The motions were recorded, and video clips were retrieved for further analysis.

### Quantitative Analysis of Subacromial Motion

**FIGURE 2:**
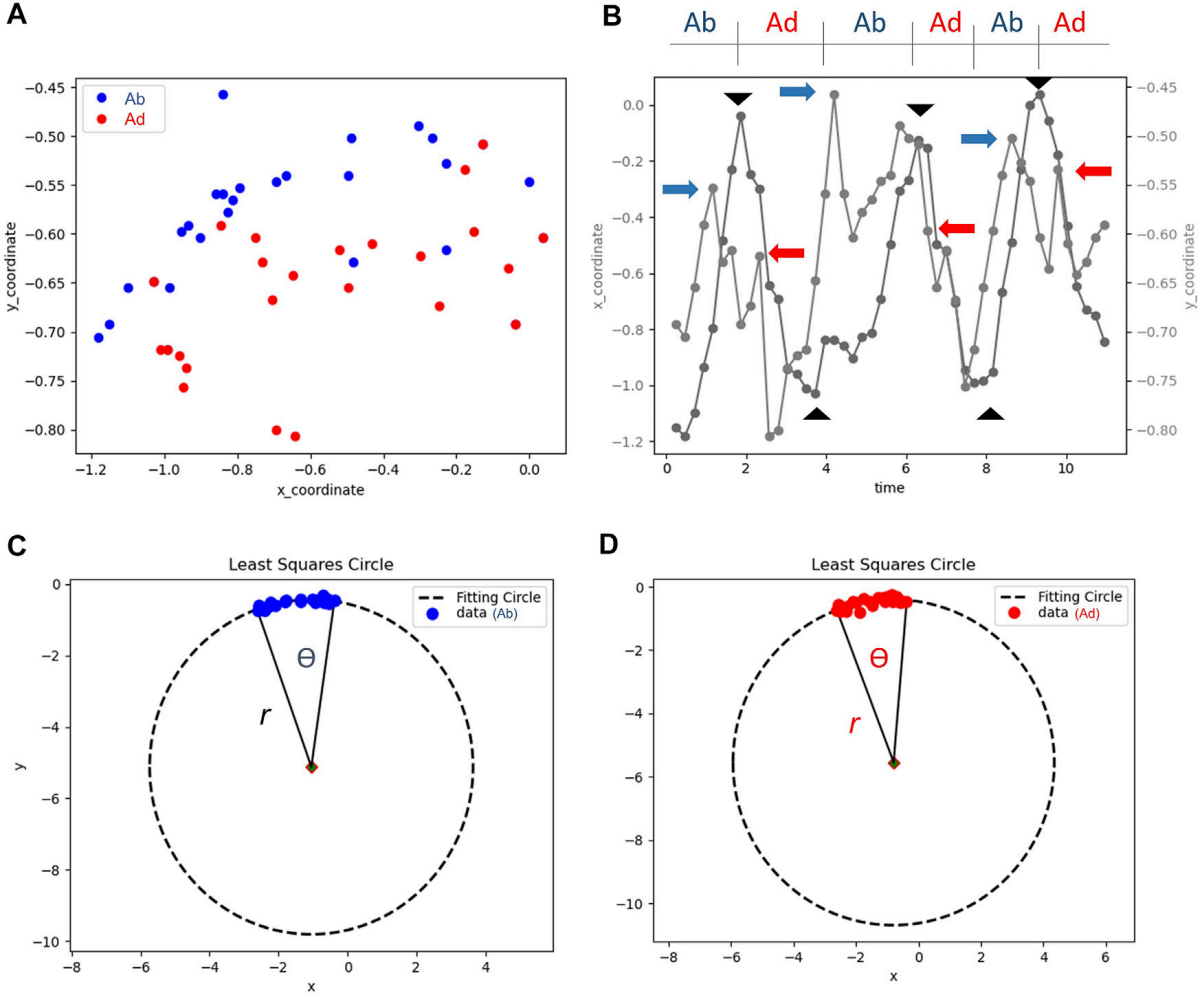
The coordinate of the greater tuberosity in relation to the lateral acromial edge during the three repetitions of arm abduction and adduction **(A)**; the location of the greater tuberostiy on the horizontal (X) and vertical (Y) axes in accordance to time **(B)**. The peaks and troughs (black arrowheads) on the trajectory of the X axis are used to define the abduction (Ab) and adduction (Ad) phases. The blue arrows in the abduction phase and the red arrows in the adduction phases indicated the points where the minimal vertical acromiohumeral distances were obtained. The locations during the abduction **(C)** and adduction **(D)** phases are fitted on a circle to calculate the rotation radius (*r*) and angle (Ѳ) of the humeral head.

The video clips were trimmed, and the middle three cycles of subacromional motion were kept for analysis. Serial images were retrieved at a rate of four frames per second. Each image was labeled sequentially on the lateral edge of the acromion and the greater tuberosity of the humeral head (Figure 1). If the greater tuberosity could not be identified from the contour of the bony cortex, we selected the most prominent point of the humeral head instead. In our computer program, the lateral edge of the acromion was designated as the reference point, and the trajectory of the greater tuberosity relative to the reference point was plotted on the X and Y axes (Figure 2A). The curve delineated on the X-axis was shown in the sine wave pattern. Each peak of the sine wave indicated the moment when the greater tuberosity rotated underneath the acromion (Figure 2B). Each wave’s trough denoted the moment when the greater tuberosity returned to its initial position. The period after the trough and before the next peak was defined as the abduction phase, whereas the period after the peak and before the next trough was defined as the adduction phase.

We identified six points on the Y-axis indicating the minimal vertical acromiohumeral distance in each phase (Figure 2B). The minimal vertical acromiohumeral distance was adapted from the common radiographic measurement of the distance between the infero-lateral edge of the acromion and the apex of the greater tuberosity of the humerus, which must be obtained during a static condition. As SIS is a syndrome occurring amid shoulder movements, we believed that our parameters acquired during dynamic motion could better reflect the patients’ clinical situations.

Values from the same abduction or adduction phases were averaged for the analysis. Furthermore, in an attempt to obtain the radius and angle of the humeral head rotation (Figures 2C,D), we used the least squares circle fitting method to fit the set of 2D points to depict the trajectory of the greater tuberosity. The goal of the least squares circle fitting method, as an optimization problem for identifying the best solution from all feasible pathways, is to minimize the cost function F defined as

$$\min\;F=\overset{n}{\underset{i=1}{\sum }}{\left(r_{i}-r_{c}\right)}^{2}$$ (1)

$$subject\;to\;r_{i}=\sqrt{{\left(x_{i}-x_{c}\right)}^{2}+{\left(y_{i}-y_{c}\right)}^{2}}\;,\;i=1,\;2,\ldots ,\;n$$ (2)

In the above formula, $x_{i}$ and $y_{i}$ are the X and Y coordinates of a 2D point describing the trajectory of the greater tuberosity, n is the total number of points, $x_{c}$ and $y_{c}$ are the X and Y coordinates of the center of the least squares circle. $r_{c}$ is the radius of the least squares circle, and the physiological meaning of $r_{c}$ is the radius of the humeral head rotation. By using the least squares circle fitting method, three output parameters ( $x_{c}$ , $y_{c}$ , and $r_{c}$ ) corresponding to the set of 2D points being fitted can be obtained.

The angle θ shown in Figures 2C,D, i.e., the angle of the humeral head rotation, was calculated using the formula:

$$\theta =\cos^{-1}\left[\frac{\left(x_{1}-x_{c}\right)\left(x_{2}-x_{c}\right)+\left(y_{1}-y_{c}\right)\left(y_{2}-y_{c}\right)}{\sqrt{{\left(x_{1}-x_{c}\right)}^{2}+{\left(y_{1}-y_{c}\right)}^{2}}\sqrt{{\left(x_{2}-x_{c}\right)}^{2}+{\left(y_{2}-y_{c}\right)}^{2}}}\right],\;and\;\;\theta <90^{{}^{\circ}}$$ (3)

where $x_{1}$ and $y_{1}$ are the X and Y coordinates of the leftmost point, and $x_{2}$ and $y_{2}$ are the X and Y coordinates of the rightmost point shown in Figures 2C,D. Thereafter, by using the least squares circle fitting method described above to fit the set of 2D points, the corresponding radius ( $r_{c}$ ) and angle ( θ ) of the humeral head rotation can be obtained. Data analysis was performed using Python (Python Software Foundation. Python Language Reference, version 3.8.3).

Furthermore, the rotation radius and angle of the humeral head were parameters specifically developed to depict dynamic subacromial motion and could not be obtained by using static shoulder ultrasound imaging. In addition, the movement of the humeral head in relation to the glenoid fossa of the scapula during arm abduction/adduction mimicked a circular trajectory. As the aforementioned parameters were estimated by the changes of the greater tuberosity’s coordinates in accordance to time, we believed that the contained clinical information would be more abundant than the acromiohumeral distance only.

### Statistical Analysis

The reliability of the quantification of subacromial motion was tested before formal enrollment. Ten video clips were recorded from the bilateral shoulders of five healthy volunteers. Quantitative analysis was performed for each video clip by the primary investigator twice, 24 h apart, to calculate the intra-rater reliability. The same measurement process was repeated by the second investigator to obtain the inter-rater reliability. The intra-class correlation coefficient (ICC) and corresponding 95% confidence interval (CI) were used to determine the reliability using the two-way mixed model. The following formula was employed to calculate the standard error of measurement (SEM): the pooled standard deviation x $\sqrt{\left(1-ICC\right)}$ . The minimal detectable change (MDC) was derived as follows:

$$MDC=1.96\times \sqrt{2}\times SEM$$ (4)

Continuous variables were expressed as mean ± standard deviation and were analyzed by Student’s t-test, analysis of variance, or Mann–Whitney U test (for non-normally distributed data). Likewise, the paired t-test or Wilcoxon signed-rank test (in case of a lack of normal distribution) was employed for univariate analysis of the correlated data. Categorical variables (presented as numbers with percentages) were analyzed using the chi-square or Fisher’s exact test (in case of sparse data). The Bland-Altman plot was employed to examine the agreement between the two measurement techniques. The generalized estimation equation (GEE) model was used to investigate the association of the quantitative measurements (dependent variables) with age, sex, shoulder laterality, body status, and differences in the shoulder motion phases and tested postures. MedCalc 14.0 (MedCalc Software, Ostend, Belgium) and SPSS 21.0 (IBM SPSS Statistics for Windows, Version 21.0, Armonk, NY, United States) were used for analysis, and a *p*-value of <0.05 was considered statistically significant.

## Results

### Basic Characteristics

**FIGURE 3:**
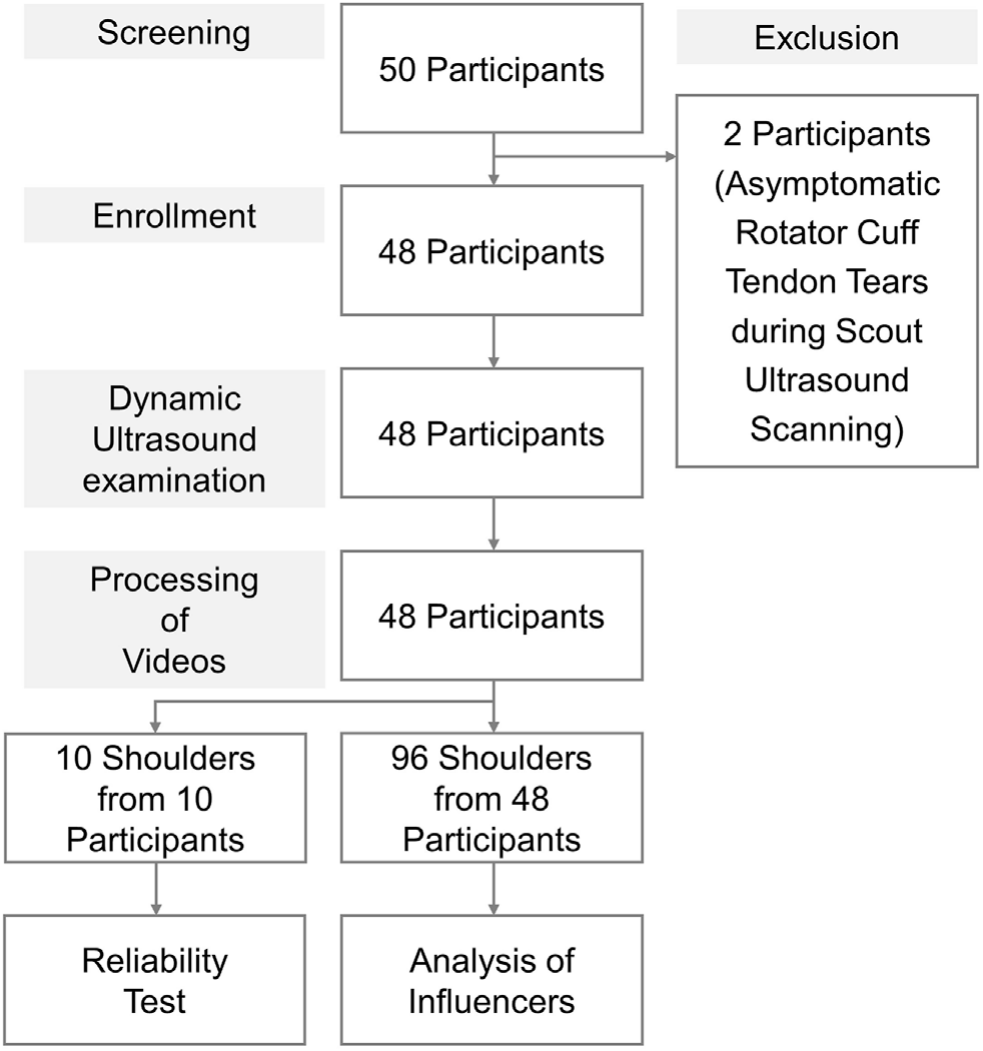
Flow diagram of participant recruitment.

A total of 48 participants were included in the present study following the exclusion of two patients with rotator cuff tendon tears. The flow diagram of participant recruitment is presented in Figure 3. Body weight and height values were higher in the male subgroups than in the age-matched female subgroups. Because we enrolled participants without shoulder complaints, the prevalence of shoulder pathologies on static ultrasound images was low, with no differences in the proportion across the subgroups (Supplementary Table S1).

### Intra-Rater and Inter-rater Reliability

Reliability was examined using the data of the minimal vertical acromiohumeral distance because the other parameters were estimated by the function for circle fitting. In terms of the intra-rater analysis, the ICC, SEM, and MDC were 0.94, 0.05, and 0.14 cm, respectively, and in terms of the inter-rater analysis, the ICC, SEM, and MDC were 0.88, 0.08, and 0.23 cm, respectively.

### Minimal Vertical Acromiohumeral Distance

In the univariate analysis, the average values in the abduction phase were significantly smaller in the adduction phase across the different subgroups (Table 1 and Supplementary Figure S1). Similarly, the values were lower values in the full-can posture than in the empty-can posture. No significant differences were identified in the comparisons between the right and left shoulders. In the GEE analysis, the abduction and full-can postures were associated with a decrease in the minimal vertical acromiohumeral distance, with a beta coefficient of −0.02 cm (95% CI, −0.03 to −0.01) and −0.07 cm (95% CI, −0.11 to −0.02), respectively (Table 2).

**TABLE 1:** Values of the minimal vertical acromiohumeral distance and rotation radius and angle of the humeral head in the different shoulder laterality, shoulder postures and motion phases.

	Right shoulder				Left shoulder			
	Full-can posture		Empty-can posture		Full-can posture		Empty-can posture	
	Abduction phase	Adduction phase	Abduction phase	Adduction phase	Abduction phase	Adduction phase	Abduction phase	Adduction phase
Minimal vertical acromiohumeral distance (cm)	0.29 ± 0.13 (0.26–0.33)	0.31 ± 0.14 (0.27–0.35)	0.38 ± 0.18 (0.33–0.44)	0.39 ± 0.19 (0.34–0.45)	0.31 ± 0.16 (0.26–0.35)	0.33 ± 0.16 (0.28–0.38)	0.37 ± 0.20 (0.31–0.43)	0.41 ± 0.21 (0.34–0.47)
Rotation radius (cm)	4.86 ± 4.11 (3.67–6.06)	6.01 ± 4.47 (4.71–7.31)	4.68 ± 2.12 (4.06–5.30)	5.36 ± 3.63 (4.31–6.42)	4.14 ± 1.86 (3.59–4.68)	6.16 ± 4.73 (4.79–7.54)	5.59 ± 5.55 (3.98–7.20)	7.77 ± 9.83 (4.92–10.63)
Rotation angle (degree)	36.03 ± 15.45 (31.54–40.52)	31.21 ± 16.87 (26.31–36.12)	36.77 ± 13.99 (32.71–40.84)	35.09 ± 14.67 (30.83–39.36)	38.76 ± 16.01 (34.11–43.41)	30.46 ± 14.55 (26.24–34.69)	39.01 ± 19.36 (33.39–44.64)	32.64 ± 17 (27.47–37.80)

Values are given as mean ± standard deviation and 95% confidence interval. The comparison of the outcome variables between the subgroups of different shoulder laterality, shoulder posture and motion phase are presented in Supplemental Figure 1-3.

**TABLE 2:** Association of the minimal vertical acromiohumeral distance, rotation radius and rotation angle of the humeral head with gender, age, body status and shoulder laterality, shoulder postures and motion phases.

	Minimal vertical acromiohumeral distance (cm)	Rotation radius (cm)	Rotation angle (degree)
Female gender	0.08 (−0.04–0.21)	−0.92 (−3.00 to 1.14)	23.83 (−11.41–59.08)
—	*p* = 0.179	*p* = 0.380	*p* = 0.185
Age (year)	<0.01 (>−0.01 to <0.01)	<0.01 (−0.02–0.03)	0.48 (0.03–0.94)
—	*p* = 0.821	*p* = 0.792	*p* = 0.034*
Height (cm)	<0.01 (>−0.01 to 0.01)	0.06 (−0.02–0.14)	1.07 (−0.40–2.55)
—	*p* = 0.390	*p* = 0.185	*p* = 0.155
Weight (kg)	<0.01 (>−0.01 to 0.01)	−0.03 (−0.10 to 0.02)	0.18 (−0.92–1.30)
—	*p* = 0.078	*p* = 0.276	*p* = 0.742
Left shoulder (right shoulder as reference)	−0.01 (−0.07 to 0.05)	0.34 (−1.00–1.69)	2.91 (−1.54–7.37)
—	*p* = 0.752	*p* = 0.614	*p* = 0.200
Full-can posture (empty-can posture as reference)	−0.07 (−0.11 to −0.02)	−0.14 (−1.26 to 0.96)	−7.51 (−15.07 to 0.03)
—	*p* = 0.002*	*p* = 0.794	*p* = 0.051
Abduction phase (adduction phase as reference)	−0.02 (−0.03 to −0.01)	−1.28 (−2.16 to −0.40)	6.60 (3.54–9.67)
—	*p* < 0.001*	*p* = 0.004*	*p* < 0.001*

The analyses were performed by using generalized estimating equations and the data were shown as the point estimates of the coefficients and their 95% confidence interval. * indicates *p* < 0.05.

### Rotation Radius of the Humeral Head

In the univariate analysis, the rotation radius of the humeral head was significantly shorter in the abduction than in the adduction phase in most of the subgroups, except for the subgroup of right shoulders in the empty-can posture (Table 1 and Supplementary Figure S2). In the GEE analysis, the abduction phase was associated with a decrease in the rotation radius, with a beta coefficient of −1.28 cm (95% CI, −2.16 to −0.40) (Table 2).

### Rotation Angle of the Humeral Head

In the univariate analysis, the rotation angle of the humeral head was significantly larger in the abduction than in the adduction phase in most of the subgroups, except for the subgroup of right shoulders in the empty-can posture (Table 1 and Supplementary Figure S3). In the GEE analysis, the abduction phase was associated with an increase in the rotation angle, with a beta coefficient of 6.60° (95% CI, 3.54–9.67) (Table 2). In addition, the increase in age was also related to a larger rotation angle, with a beta coefficient of 0.48° (95% CI, 0.03–0.94).

The rotation angle of the humeral head in the abduction phase was assessed in comparison with the actual angle of arm abduction measured by the smartphone application. Significant discrepancies between the angle measurements were observed in all subgroups, with mean differences ranging from 5.10° to 12.40°, as shown in the Bland-Altman plot (Supplementary Figure S4).

### Correlation Analysis

A significant negative correlation was observed in all subgroups between the rotation angle and radius of the humeral head (Supplementary Figure S5) and between the rotation angle of the humeral head and the minimal vertical acromiohumeral distance (Supplementary Figure S6). On the other hand, a significant positive correlation was observed in some subgroups between the radius of humeral head rotation and minimal vertical acromiohumeral distance (Supplementary Figure S7).

## Discussion

This study aimed to delineate subacromial motions using quantitative dynamic ultrasound, contributing to several important findings. First, the intra-rater and inter-rater reliabilities of this imaging method were acceptable, allowing comparisons of the parameters among different individuals. Second, a decrease in the minimal vertical acromiohumeral distance was observed in the abduction phase and full-can posture. Third, the abduction phase was associated with a shorter rotation radius and a larger rotation angle of the humeral head. Fourth, a significant discrepancy was identified between the rotation angle of the humeral head and the actual angle of arm abduction.

The acomiohumeral distance is the most common sonographic parameter for the evaluation of SIS. In 2018, Kozono et al. assessed 11 patients with rotator cuff tendon tears and 10 healthy controls and reported a decrease in the acomiohumeral distance in the patient group (Kozono et al., 2018). In 2020, de Oliveira et al. investigated 45 recreational athletes and found that the acomiohumeral distance did not decrease in the painful shoulders compared to the asymptomatic shoulders (de Oliveira et al., 2019). As the acomiohumeral distance reported by the antecedent studies was calculated while the shoulders were maintained at a fixed degree of abduction, the data might not be relevant for disorders that occur only during motion. In this regard, Bureau et al. developed a semi-quantitative method to grade SIS during shoulder elevation: Grade 0, no impingement; Grade 1, pain with no soft tissue impingement; Grade 2, pain with soft tissue impingement; and Grade 3, pain with upward migration of the humeral head (Bureau et al., 2006). Using the aforementioned methods, Chang et al. demonstrated the association between effusion encircling the long head of the bicep tendon and different severities of impingement (Chang et al., 2016) with the predictability of the dynamic sonographic results for the effectiveness of ultrasound-guided subacromial injection (Chang et al., 2017). However, the grading system is based on the examiner’s perception of subacromial motion and soft tissue deformation, which may sometimes lead to disagreement among different investigators. Therefore, the main strength of our method lies in the retrieval of quantitative data of the humeral head trajectory.

Our study revealed that the shoulders in the abduction phase had a smaller vertical minimal acromiohumeral distance than that of shoulders in the adduction phase. During the abduction phase, the concentric contraction of the deltoid and supraspinatus muscles exerts an upward and medially deviated force on the humeral head, which brings the humeral center of rotation in proximity to the acromion and scapular glenoid cavity. On the other hand, during the adduction phase, the deltoid and supraspinatus muscles are elongated owing to eccentric contraction, and the gravity pulls the humeral center of rotation downward. The findings also indicated that our protocol was capable of delineating differences in the vertical acromiohumeral distance due to variations in the muscle activation patterns between the abduction and adduction phases. Furthermore, compared with the empty-can posture, the full-can posture was associated with a decrease in the vertical minimal acromiohumeral distance. In the full-can posture, the arm is externally rotated, and the most prominent portion of the greater tuberosity is brought underneath the acromion arc, leading to a narrower subacromial space. A previous MRI study investigating 18 asymptomatic shoulders confirmed that the footprint of the humeral head was at the highest risk of subacromial impingement at 30% of the external rotation cycle of the arm (Coats-Thomas et al., 2018). Our results imply that the full-can posture might serve as a better stress position than the empty-can posture in reproducing SIS during dynamic ultrasound examinations.

**FIGURE 4:**
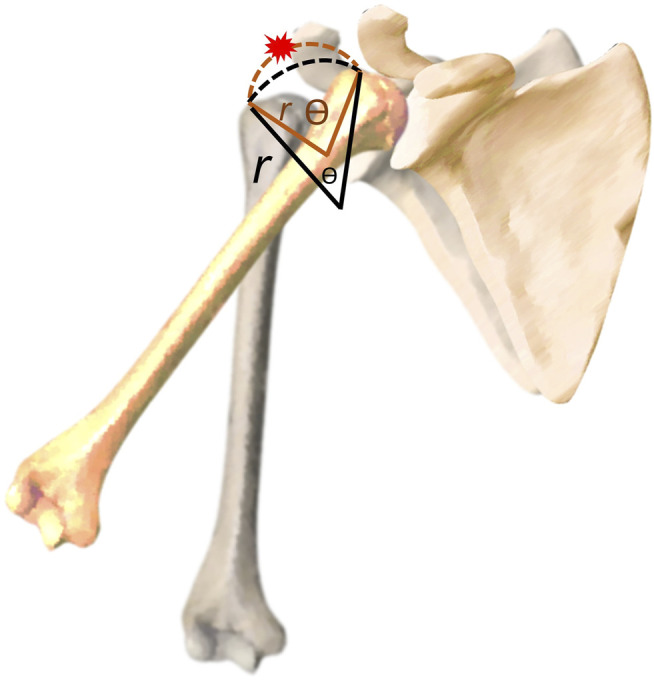
Illustration of the association between the rotation angle (Ѳ) and radius (*r*) and why a bigger angle is associated with an increased risk of subacromial impingement.

The rotation radius and angle of the humeral head are the parameters based on the trajectory of the greater tuberosity, which have never been used in any existing studies. If the straight distance of the greater tuberosity from the starting point to the subacromial region is assumed to be the same, a decrease in the rotation radius would be associated with an increase in the rotation angle. A larger rotation angle may imply that more efforts are needed to transition the greater tuberosity from the starting position to the undersurface of the acromion, possibly owing to subacromial impingement (Figure 4). Our theory is supported by the correlation analysis, which revealed that an increased rotation angle leads to a narrower vertical acromiohumeral distance. Furthermore, our techniques can be incorporated with artificial intelligence (Cheng and Malhi, 2017; Tsai et al., 2020) in the future to depict the sub-acromial motion in a speedy manner.

In addition, the angle of arm abduction (measured by the smartphone application) was mostly larger than the rotation angle of the humeral head (estimated by dynamic ultrasound), with a mean difference ranging from 5.10° to 12.40° across the subgroups (Supplementary Figure S4). As the rotation angle of the humeral head was estimated by the reciprocal movement between the greater tuberosity and lateral acromial edge, the degree of scapular rotation was neglected. Therefore, the discrepancy between both angles might be used as an indirect indicator of scapular rotation, which enables better delineation of the scapular thoracic rhythm.

In the present study, GEE was used to adjust for potential confounders on the ultrasound parameters. The increase in age was related to a larger rotation angle but its beta coefficient analyzed by GEE was really small (0.48°, 95% CI, 0.03–0.94). Because age, sex, and body status had minimal impact on all the ultrasound parameters, it is possible to apply this method to the evaluation of subacromial motions in the general population.

There are several limitations to this study that need to be acknowledged. First, ultrasound imaging cannot visualize the structures underneath the body cortex. Therefore, our method could not be applied to the evaluation of internal impingement between the humeral head and the bony glenoid. Second, as this was a pilot study examining the reliability and potential confounders of several new parameters, we included only healthy participants. In the future, a prospective trial needs to be conducted to assess its usefulness in patients with SIS. Third, a smartphone was attached to the middle arm during the dynamic tests. Although the weight of the device is less than 150 g, the loaded condition might have influenced the shoulder kinematics and affected the outcomes. Fourth, the thickness of the deltoid muscle and supraspinatus tendon as well as the shape of the humeral head and acromion could possibly influence the parameters of dynamic ultrasound imaging. Interpretation for subacromial motion abnormality should be cautious by considering the difference in the aforementioned structures on each individual. Fifth, the subacromial-subdeltoid bursa is prone to be impinged during shoulder abduction, which played a substantial role in the development of SIS. However, none of the included participants had subdeltoid bursitis because all of them were asymptomatic for shoulder pain. It will be of clinical interest to know how the dynamic ultrasound parameters change in patients with thickened subacromial-subdeltoid bursa, which has been served as our on-going research focus.

## Conclusion

Quantitative analysis of dynamic ultrasound imaging allows the delineation of subacromial motion with good reliability. The vertical acromiohumeral distance was the lowest in the abduction and full-can postures. The rotation angle of the humeral head was negatively correlated with the vertical acromiohumeral distance and has the potential to serve as a new parameter for evaluating SIS. Further prospective studies are needed to evaluate the usefulness of the imaging method in patients with shoulder pain.

## Data Availability

The original contributions presented in the study are included in the article/Supplementary Material, further inquiries can be directed to the corresponding author.

## Abbreviations

CI: confidence interval; GEE: generalized estimation equation; ICC: intra-class correlation coefficient; MDC: minimal detectable change; MRI: magnetic resonance imaging; SEM: standard error of measurement; SIS: subaromial impingement syndrome; SPADI: shoulder pain and disability index.

## References

[B1] Bureau N. J. Beauchamp M. Cardinal E. Brassard P. (2006). Dynamic Sonography Evaluation of Shoulder Impingement Syndrome. Am. J. Roentgenology 187 (1), 216–220. 10.2214/AJR.05.0528 16794179

[B2] Chang K.-V. Wu W.-T. Chen M.-C. Chiu Y.-C. Han D.-S. Chen C.-C. (2019). Smartphone Application with Virtual Reality Goggles for the Reliable and Valid Measurement of Active Craniocervical Range of Motion. Diagnostics 9 (3), 71. 10.3390/diagnostics9030071 PMC6787724 31295869

[B3] Chang K.-V. Wu W.-T. Han D.-S. Özçakar L. (2017). Static and Dynamic Shoulder Imaging to Predict Initial Effectiveness and Recurrence after Ultrasound-Guided Subacromial Corticosteroid Injections. Arch. Phys. Med. Rehabil. 98 (10), 1984–1994. 10.1016/j.apmr.2017.01.022 28245972

[B4] Chang K.-V. Wu W.-T. Hsu P.-C. Lew H. L. Özçakar L. (2020a). Clinical Tests of the Shoulder. Am. J. Phys. Med. Rehabil. 99 (2), 161–169. 10.1097/PHM.0000000000001311 31584452

[B5] Chang K.-V. Wu W.-T. Özçakar L. (2016). Association of Bicipital Peritendinous Effusion with Subacromial Impingement: A Dynamic Ultrasonographic Study of 337 Shoulders. Sci. Rep. 6, 38943. 10.1038/srep38943 27941908 PMC5150575

[B6] Chang P.-H. Chen Y.-J. Chang K.-V. Wu W.-T. Özçakar L. (2020b). Ultrasound Measurements of Superficial and Deep Masticatory Muscles in Various Postures: Reliability and Influencers. Sci. Rep. 10 (1), 14357. 10.1038/s41598-020-71378-z 32873849 PMC7463001

[B7] Cheng P. M. Malhi H. S. (2017). Transfer Learning with Convolutional Neural Networks for Classification of Abdominal Ultrasound Images. J. Digit Imaging 30 (2), 234–243. 10.1007/s10278-016-9929-2 27896451 PMC5359213

[B8] Coats-Thomas M. S. Massimini D. F. Warner J. J. P. Seitz A. L. (2018). *In Vivo* Evaluation of Subacromial and Internal Impingement Risk in Asymptomatic Individuals. Am. J. Phys. Med. Rehabil. 97 (9), 659–665. 10.1097/phm.0000000000000940 29613881

[B9] Cunningham G. Nicodème-Paulin E. Smith M. M. Holzer N. Cass B. Young A. A. (2018). The Greater Tuberosity Angle: a New Predictor for Rotator Cuff Tear. J. Shoulder Elbow Surg. 27 (8), 1415–1421. 10.1016/j.jse.2018.02.051 29703680

[B10] de Oliveira F. C. L. Ager A. L. Roy J.-S. (2021). Is There a Decrease in the Acromiohumeral Distance Among Recreational Overhead Athletes with Rotator Cuff-Related Shoulder Pain? J. Sport Rehabil. 30, 531–537. 10.1123/jsr.2020-0170 33120355

[B11] de Oliveira F. C. L. Pairot de Fontenay B. Bouyer L. J. Roy J.-S. (2019). Immediate Effects of Kinesiotaping on Acromiohumeral Distance and Shoulder Proprioception in Individuals with Symptomatic Rotator Cuff Tendinopathy. Clin. Biomech. 61, 16–21. 10.1016/j.clinbiomech.2018.11.005 30453120

[B12] Han D.-S. Wu W.-T. Hsu P.-C. Chang H.-C. Huang K.-C. Chang K.-V. (2021). Sarcopenia Is Associated with Increased Risks of Rotator Cuff Tendon Diseases Among Community-Dwelling Elders: A Cross-Sectional Quantitative Ultrasound Study. Front. Med. 8 (566), 630009. 10.3389/fmed.2021.630009 PMC8131871 34026779

[B13] Kenmoku T. Matsuki K. Ochiai N. Sonoda M. Ishida T. Sasaki S. (2019). Comparison of Glenohumeral Joint Rotation between Asymptomatic Subjects and Patients with Subacromial Impingement Syndrome Using Cine-Magnetic Resonance Imaging: a Cross-Sectional Study. BMC Musculoskelet. Disord. 20 (1), 475. 10.1186/s12891-019-2818-3 31653240 PMC6815044

[B14] Kozono N. Okada T. Takeuchi N. Hamai S. Higaki H. Shimoto T. (2018). *In Vivo* dynamic Acromiohumeral Distance in Shoulders with Rotator Cuff Tears. Clin. Biomech. 60, 95–99. 10.1016/j.clinbiomech.2018.07.017 30340151

[B15] Lo Y. P. Hsu Y. C. Chan K. M. (1990). Epidemiology of Shoulder Impingement in Upper Arm Sports Events. Br. J. Sports Med. 24 (3), 173–177. 10.1136/bjsm.24.3.173 2078803 PMC1478773

[B16] Roy J. S. Braën C. Leblond J. Desmeules F. Dionne C. E. MacDermid J. C. (2015). Diagnostic Accuracy of Ultrasonography, MRI and MR Arthrography in the Characterisation of Rotator Cuff Disorders: a Systematic Review and Meta-Analysis. Br. J. Sports Med. 49 (20), 1316–1328. 10.1136/bjsports-2014-094148 25677796 PMC4621376

[B17] Tsai J. Y. Jan Y.-K. Liau B.-Y. Subiakto R. B. R. Lin C.-Y. Hendradi R. (2020). “A Convolutional Neural Network Model to Classify the Effects of Vibrations on Biceps Muscles,” in Advances in Physical, Social and Occupational Ergonomics. AHFE 2020. Advances in Intelligent Systems and Computing. Editors Karwowski W. Goonetilleke R. Xiong S. Goossens R. Murata A., Vol. 1215 (Springer, Cham). 10.1007/978-3-030-51549-2_8

[B18] van der Windt D. A. Koes B. W. de Jong B. A. Bouter L. M. (1995). Shoulder Disorders in General Practice: Incidence, Patient Characteristics, and Management. Ann. Rheum. Dis. 54 (12), 959–964. 10.1136/ard.54.12.959 8546527 PMC1010060

[B19] Wu W.-T. Chen L.-R. Chang H.-C. Chang K.-V. Özçakar L. (2021). Quantitative Ultrasonographic Analysis of Changes of the Suprascapular Nerve in the Aging Population with Shoulder Pain. Front. Bioeng. Biotechnol. 9, 640747. 10.3389/fbioe.2021.640747 33681173 PMC7933457

[B20] Yao M. Yang L. Cao Z.-y. Cheng S.-d. Tian S.-l. Sun Y.-l. (2017). Translation and Cross-Cultural Adaptation of the Shoulder Pain and Disability Index (SPADI) into Chinese. Clin. Rheumatol. 36 (6), 1419–1426. 10.1007/s10067-017-3562-4 28191606

