# Supplementary material for: Quantitative Analysis of Dynamic Subacromial Ultrasonography: Reliability and Influencing Factors
Source: Front Bioeng Biotechnol. 2022 Feb 15;10:830508. doi: 10.3389/fbioe.2022.830508 (PMC8886165; doi:10.3389/fbioe.2022.830508)
Supplement: Supplementary file 1 [file DataSheet1.docx]

**Quantitative Analysis of Dynamic Subacromial Ultrasonography: Reliability and Influencing Factors**

**Supplemental Material:**

Supplemental Table 1

Supplemental Figure 1-7

**Supplemental Table 1.** Characteristics and sonographic findings of the 48 participants

|  | **≥20 to <40 years in age** | |  | **≥40 to <60 years in age** | |  | **≥60 years in age** | |  |  |
| --- | --- | --- | --- | --- | --- | --- | --- | --- | --- | --- |
|  | Young  Men | Young  Women |  | Middle-aged  Men | Middle-aged  Women |  | Old  Men | Old  Women |  | *p* value  (overall) |
| **Participants’ characteristics** |  |  |  |  |  |  |  |  |  |  |
| Age (year) | 32.57 ± 6.02  (27.00 to 38.14) | 30.28 ± 5.52  (25.17 to 35.39) |  | 48.11 ± 4.67  (44.51 to 51.70) | 46.33 ± 5.08  (40.99 to 51.67) |  | 69.12 ± 4.94  (64.99 to 73.25) | 69.72 ± 4.92  (66.42 to 73.03) |  | <0.001* |
| Height (cm) | 174.38 ± 5.49  (169.30 to 179.46) | 161.00 ± 5.38  (154.31 to 167.68) |  | 175.50 ± 5.07  (170.80 to 180.19) | 159.00 ± 4.42  (154.35 to 163.64) |  | 167.81 ± 8.25  (160.18 to 175.44) | 155.53 ± 6.21  (150.34 to 160.73) |  | <0.001* |
| Weight (kg) | 71.92 ± 6.44  (65.96 to 77.88) | 54.60 ± 7.92  (44.76 to 64.43) |  | 78.97 ± 9.39  (70.28 to 87.65) | 55.00 ± 3.16  (51.68 to 58.31) |  | 68.00 ± 9.65  (59.06 to 76.93) | 52.05 ± 6.47  (46.63 to 57.46) |  | <0.001* |
| **Shoulder pathology** |  |  |  |  |  |  |  |  |  |  |
| Biceps tendinopathy | 0 | 0 |  | 0 | 0 |  | 0 | 0 |  | N.A. |
| Subscapularis tendinopathy | 0 | 0 |  | 0 | 0 |  | 0 | 0 |  | N.A. |
| Subscapularis calcification | 0 | 0 |  | 1 | 0 |  | 0 | 1 |  | 0.704 |
| Supraspinatus tendinopathy | 0 | 0 |  | 0 | 0 |  | 0 | 0 |  | N.A. |
| Supraspinatus calcification | 0 | 0 |  | 0 | 0 |  | 1 | 0 |  | 0.403 |
| Subacromial-subdeltoid bursitis | 0 | 0 |  | 0 | 0 |  | 0 | 0 |  | N.A. |
| Infraspinatus tendinopathy | 0 | 0 |  | 0 | 0 |  | 0 | 0 |  | N.A. |
| Infraspinatus calcification | 0 | 0 |  | 0 | 0 |  | 1 | 0 |  | 0.403 |

The continuous data are shown as mean ± standard deviation and the categorical variables are shown as number. N.A, not applicable due to zero count in each selected cell.

**Supplementary Figure legends**


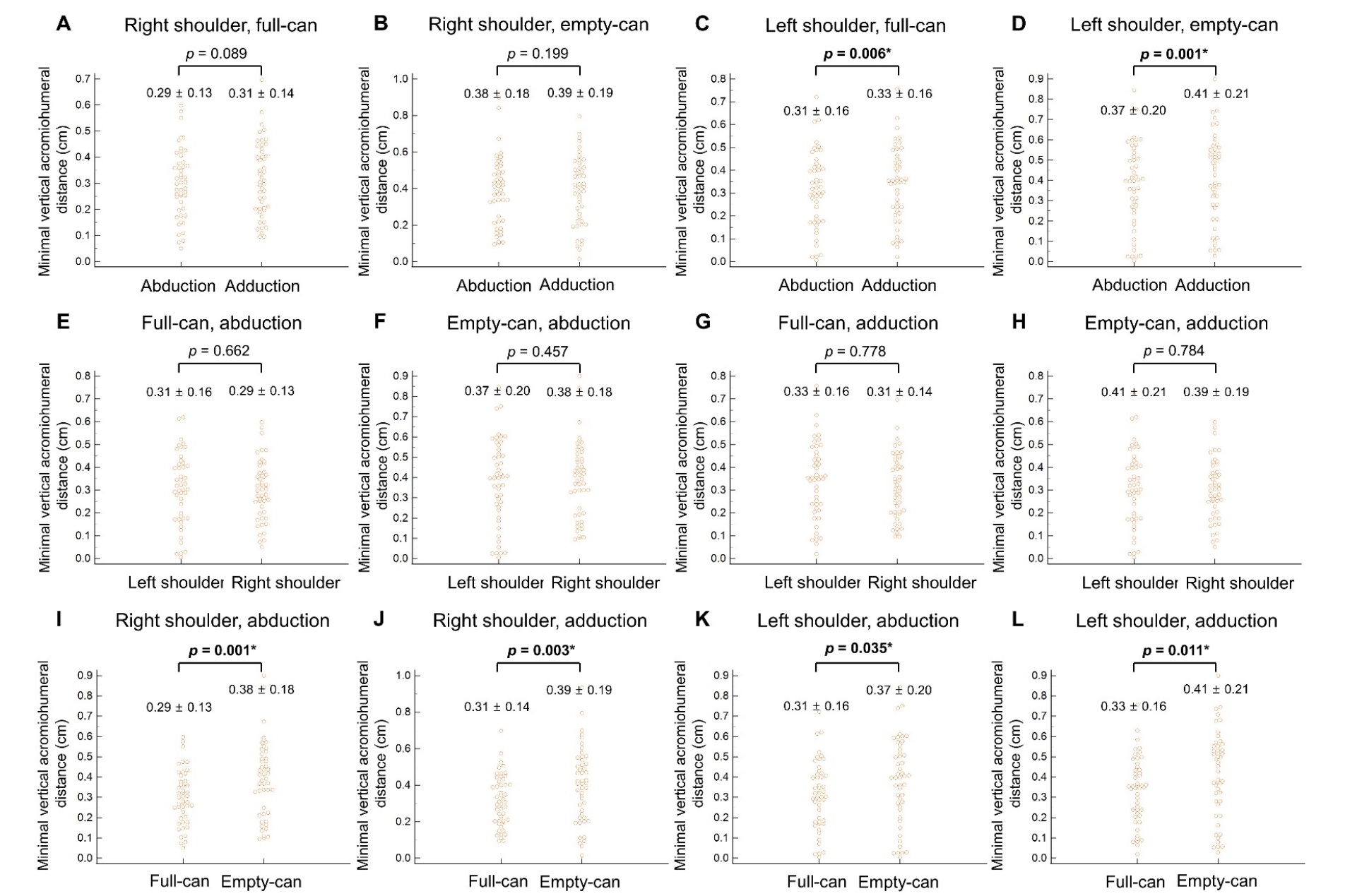


**Supplementary Figure 1.** Comparison of the minimal vertical acromiohumeral distance between the subgroups with different shoulder laterality, postures (full-can vs. empty-can) and phases (abduction vs. adduction).


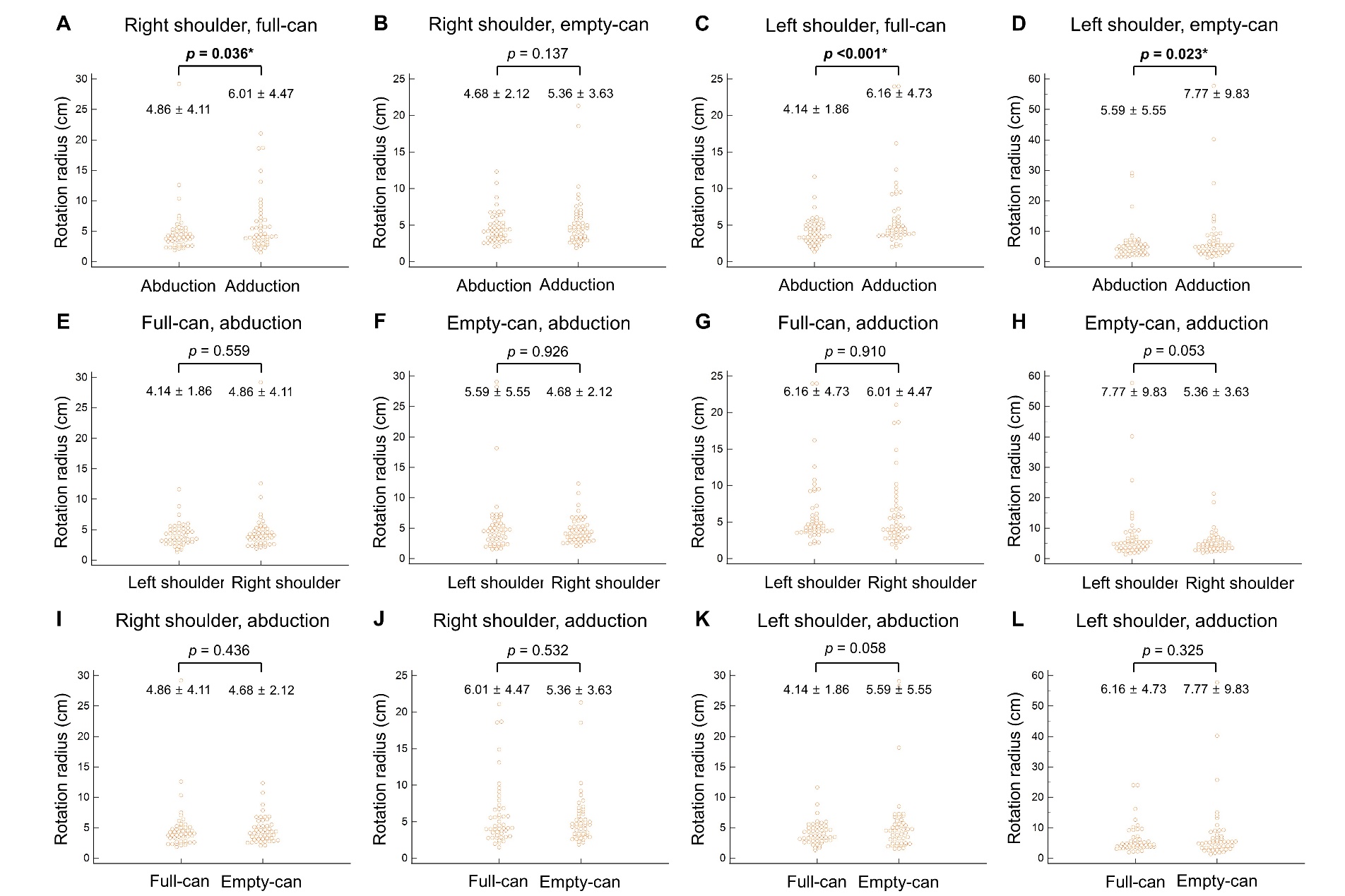


**Supplementary Figure 2.** Comparison of the rotation radius of the humeral head between the subgroups with different shoulder laterality, postures (full-can vs. empty-can) and phases (abduction vs. adduction).


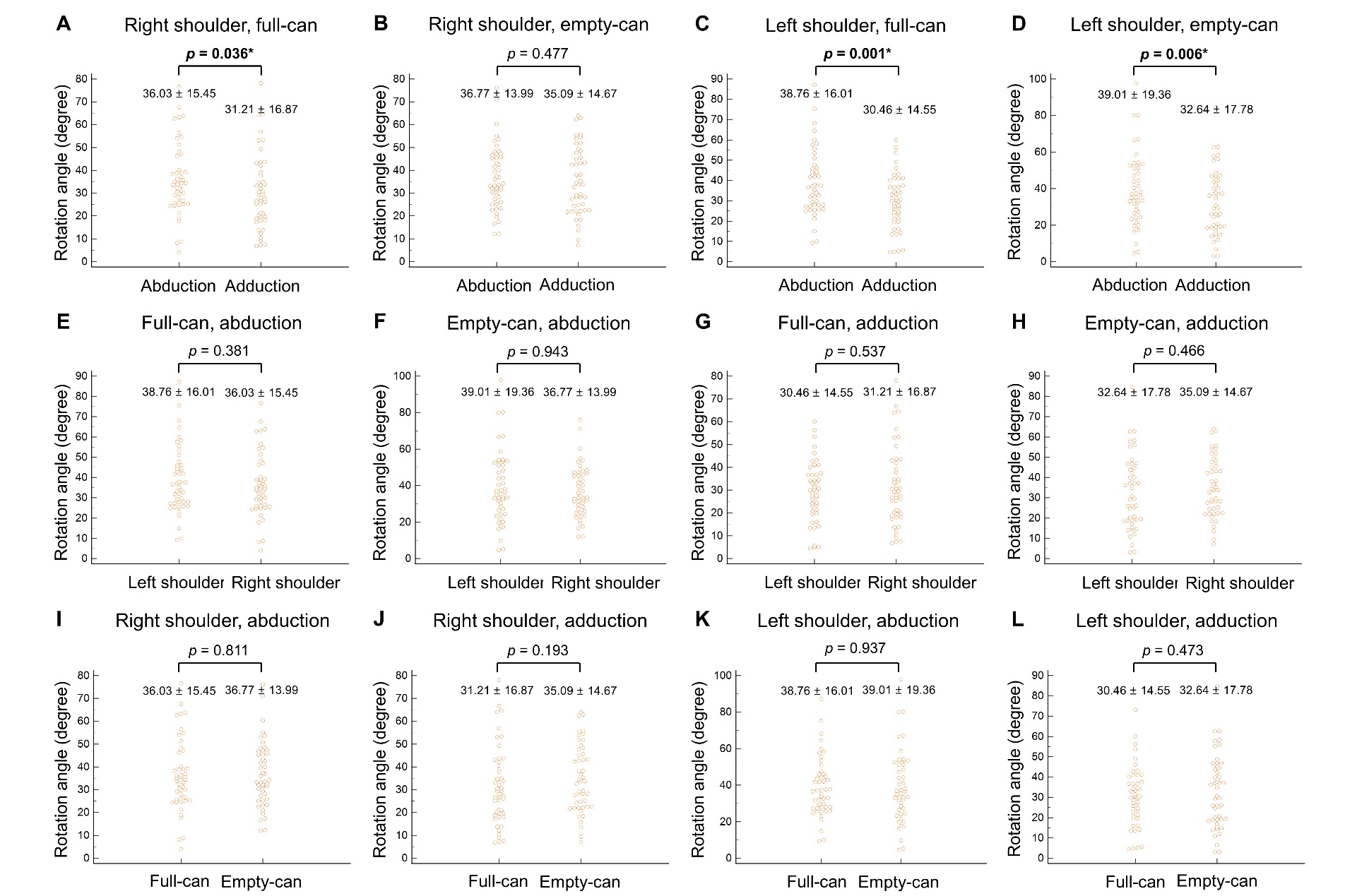


**Supplementary Figure 3.** Comparison of the rotation angle of the humeral head between the subgroups with different shoulder laterality, postures (full-can vs. empty-can) and phases (abduction vs. adduction).


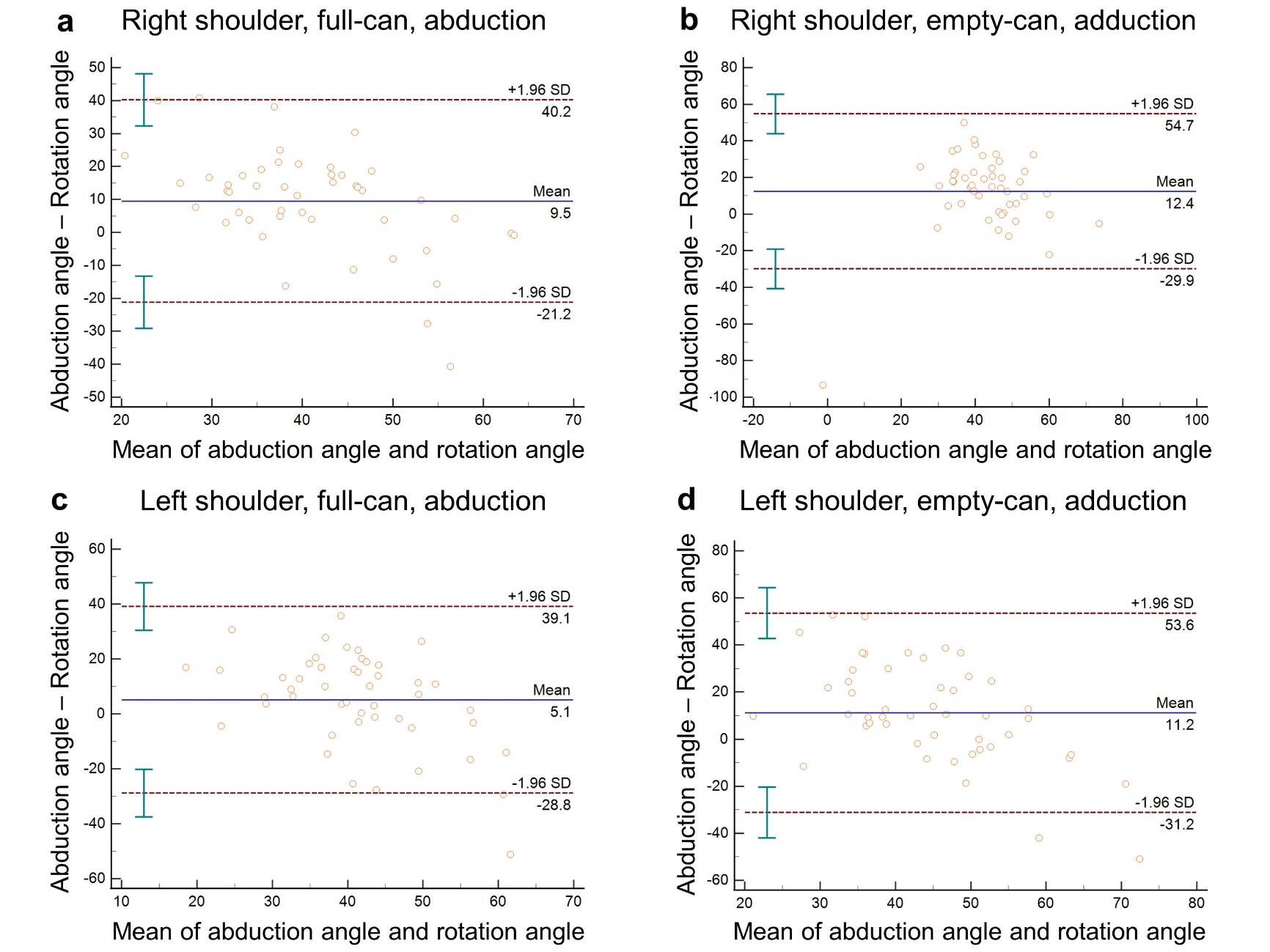


**Supplementary Figure 4.** The Bland–Altman plot for examining the consistency between the actual angle of arm abduction measured by the smartphone application and the rotation angle of the humeral head in the abduction phase. The blue solid line indicates the mean values of all the measurements, whereas the red dotted line refers to its upper and lower limits (mean ± 1.96 standard deviation).


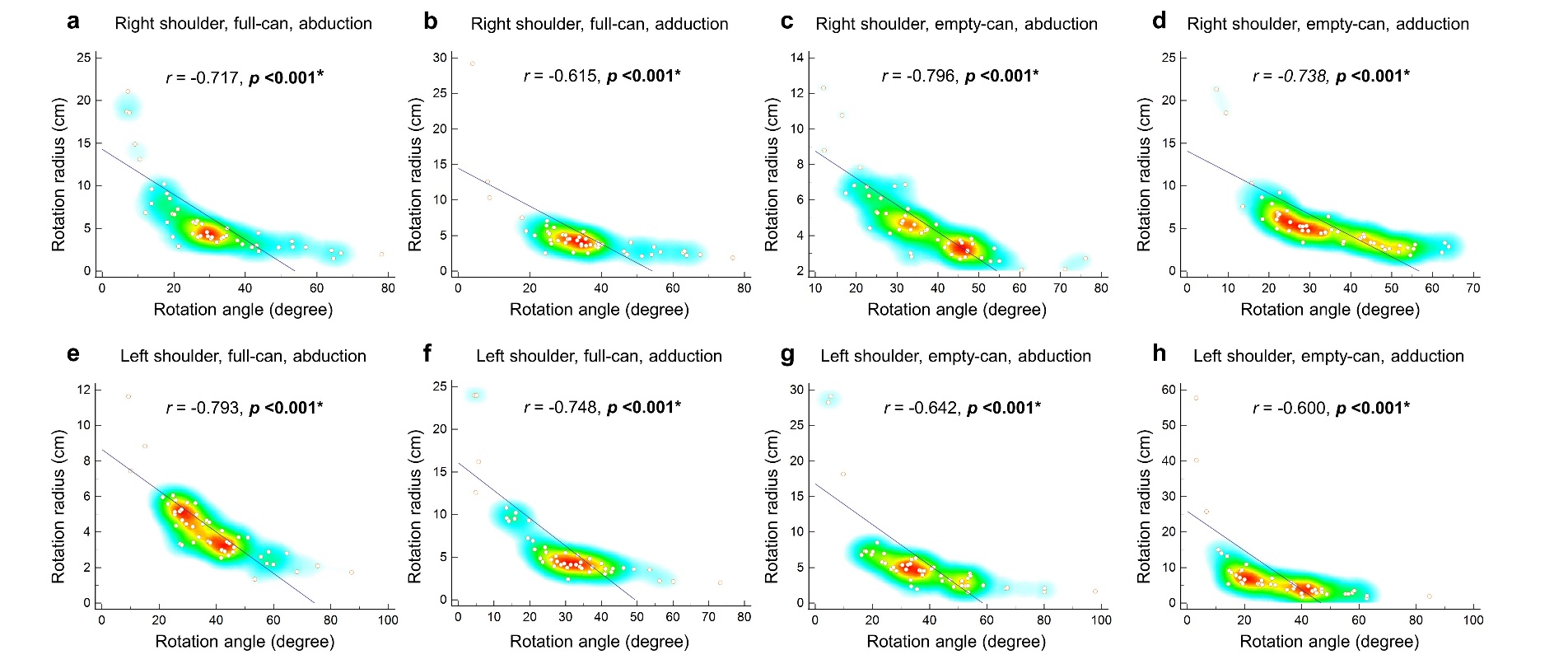


**Supplementary Figure 5.** Correlation between the rotation radius and angle of the humeral head in the subgroups with different shoulder laterality, postures (full-can vs. empty-can) and phases (abduction vs. adduction). The regression line is plotted on when *p* value is less than 0.05. The heat map with background color coding suggests clusters of observations.


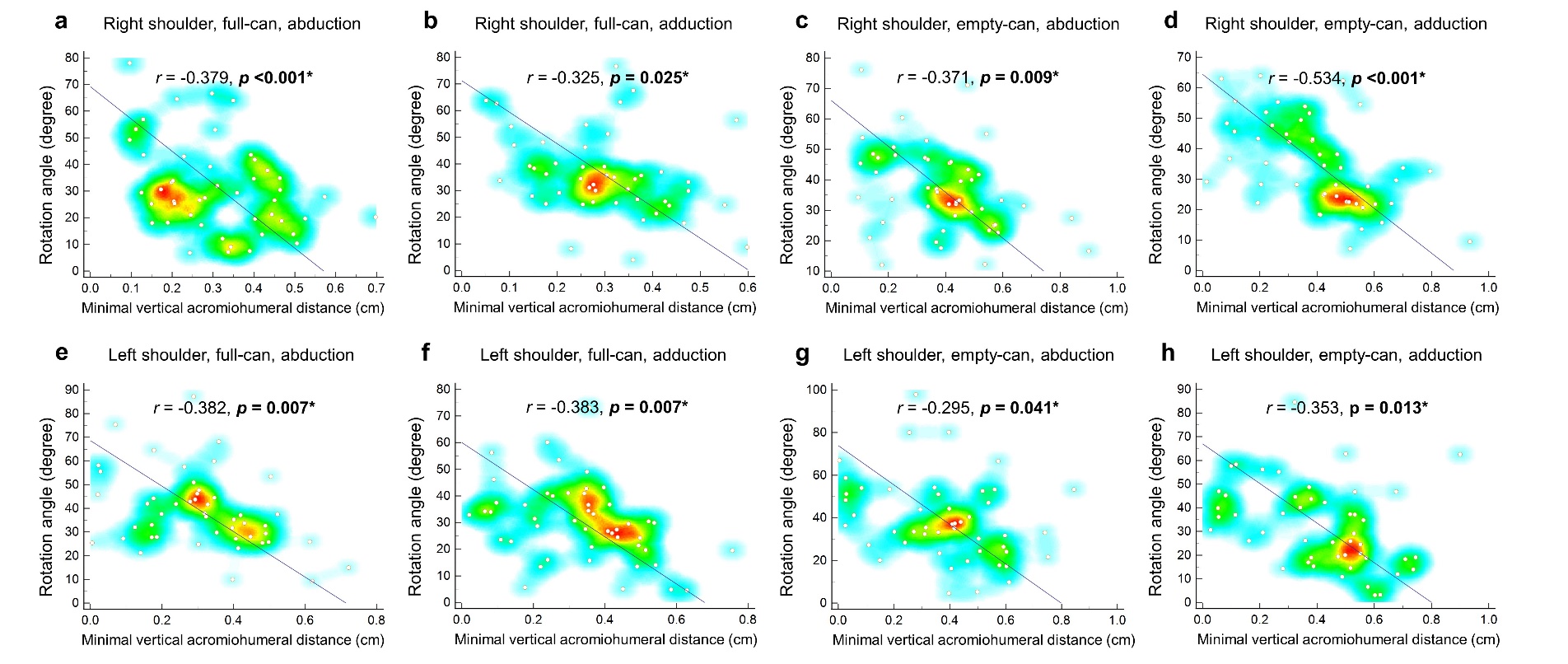


**Supplementary Figure 6.** Correlation between the rotation angle of the humeral head and minimal vertical acromiohumeral distance in the subgroups with different shoulder laterality, postures (full-can vs. empty-can) and phases (abduction vs. adduction). The regression line is plotted on when *p* value is less than 0.05. The heat map with background color coding suggests clusters of observations.


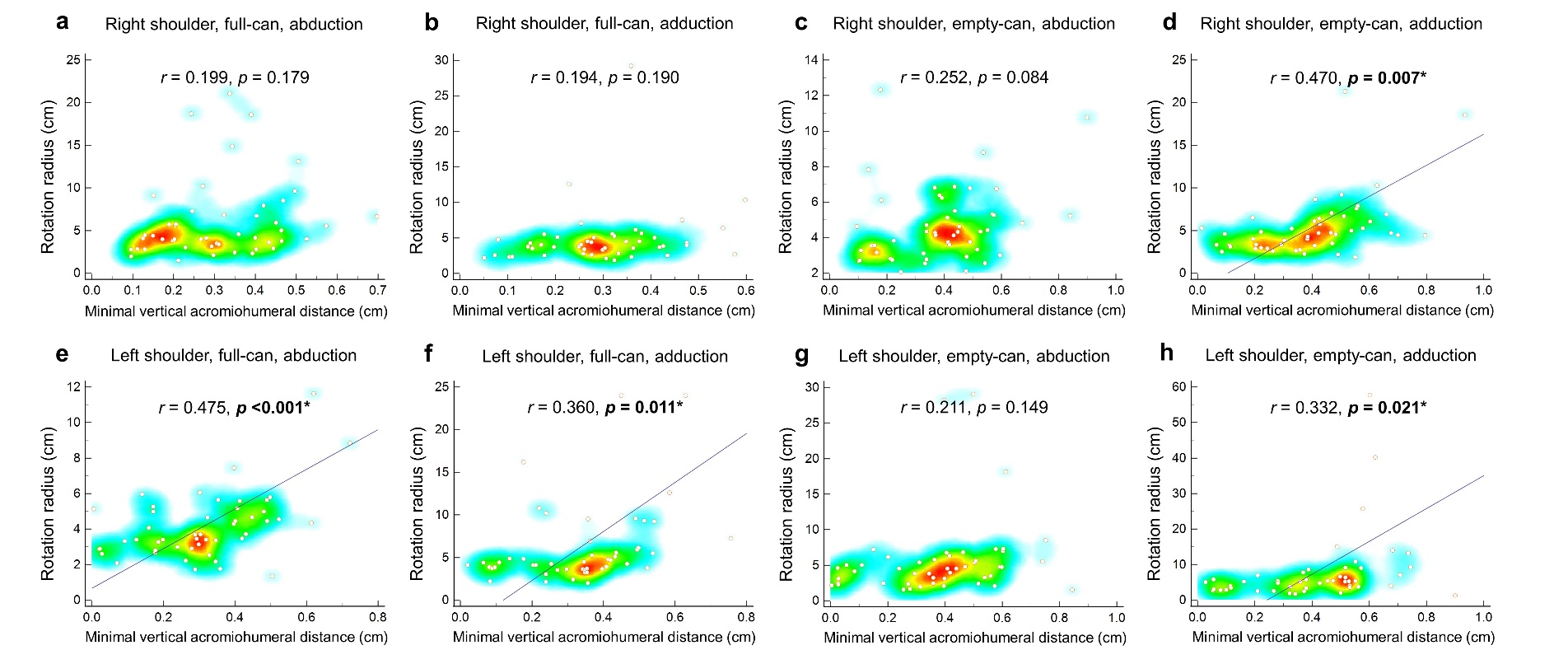


**Supplementary Figure 7.** Correlation between the rotation radius of the humeral head and minimal vertical acromiohumeral distance in the subgroups with different shoulder laterality, postures (full-can vs. empty-can) and phases (abduction vs. adduction). The regression line is plotted on when *p* value is less than 0.05. The heat map with background color coding suggests clusters of observations.
